# “Why don’t I look like her?” How adolescent girls view social media and its connection to body image

**DOI:** 10.1186/s12905-022-01845-4

**Published:** 2022-06-27

**Authors:** Alana Papageorgiou, Colleen Fisher, Donna Cross

**Affiliations:** 1 Telethon Kids Institute, University of Western Australia, PO Box 855, West Perth, WA 6872 Australia; 2 School of Population and Global Health, University of Western Australia, Perth, Australia; 3 Edith Cowan University, Perth, Australia

**Keywords:** Adolescence, Instagram, Body image, Sexualization, Appearance comparisons, Self-objectification

## Abstract

**Background:**

Adolescent girls appear more vulnerable to experiencing mental health difficulties from social media use than boys. The presence of sexualized images online is thought to contribute, through increasing body dissatisfaction among adolescent girls. Sexual objectification through images may reinforce to adolescent girls that their value is based on their appearance. This study explored how sexualized images typically found on social media might influence adolescent girls’ mental health, in positive and/or negative ways.

**Methods:**

In-depth interviews were conducted with girls aged 14–17 years (n = 24) in Perth, Western Australia. Data were analyzed using thematic analysis.

**Results:**

Participants identified body image as a major concern, reporting negative appearance comparisons when viewing images on social media. Appearance comparisons were perceived to exacerbate adolescent girls’ appearance-based concerns. Comparisons also influenced adolescent girls’ efforts to change their appearance and seek validation on social media. The importance of awareness and education from a younger age about social media and its influence on body image was emphasized, as was the need for strategies to promote positive body image and counteract negative body image.

**Conclusion:**

The findings of this study have important implications for professionals working with adolescent girls and for the development of health promotion programs addressing social media use and body image concerns.

**Supplementary Information:**

The online version contains supplementary material available at 10.1186/s12905-022-01845-4.

## Background

Adolescence is an important period of development, with major physical, social, cognitive and emotional changes, and identity formation occurring [1]. Adolescence is also a time when young people begin to use social media, online platforms enabling social interaction through the creation of individualized online profiles and sharing of photos, videos and other media on sites or apps such as Instagram, Snapchat and Facebook [2, 3]. Social media has been found to have both positive and negative impacts on the lives of adolescents. Positive aspects of social media use include increased peer connection and support, and opportunities to learn [4–6]. However, research has largely reported adverse influences from adolescents’ social media use, contributing to mental health difficulties including increased depression, anxiety, and self-harm behaviors, decreased socio-emotional wellbeing, low self-esteem and negative body image [7–10]. For girls, the combination of reaching puberty, their body changing, and the importance of approval from peers and romantic relationship formation can increase vulnerability to negative body image and research suggests social media may have a greater influence on their body dissatisfaction compared to boys’ [2, 11, 12].

Body image encompasses the thoughts, feelings, beliefs and attitudes one has about their body and appearance [13]. Body dissatisfaction is an important element of body image and can range in severity from having a preference for different body characteristics to the uptake of extreme action to change one’s body [14]. Body dissatisfaction has been linked to low self-esteem, decreased mental health and wellbeing, and the development of eating disorders among adolescent girls [14–17]. The potential harms associated with body dissatisfaction highlight negative body image as an important public health concern [18].

Time spent on the Internet has been associated with increased body dissatisfaction among adolescent girls, with the interaction allowed by social media and appearance-focused content influencing body image concerns through negative social comparisons and peer normative processes [11, 19–22]. Images of attractive thin females, often photo-shopped with filters, feature frequently on social media platforms such as Instagram, promoting stereotyped beauty ideals subsequently affecting viewers’ body image and dissatisfaction [9, 23]. The females in images on social media are more commonly peers rather than celebrities like those included in mass media, which may influence body image related attitudes and concerns more significantly, given peers’ relatability and relevance to girls’ daily lives [19, 24, 25]. A study investigating the effect of manipulated Instagram selfies on adolescent girls’ body image found such images resulted in poorer body image perception, especially among those with high levels of social comparison [24]. Given the increasing prevalence of image-sharing online, young people may need support to improve their self-esteem and become more informed consumers of digital images (e.g. being able to identify enhanced or photo-shopped images as unrealistic and unattainable).

While social media can also counteract negative body image messages with positive body image accounts, even these accounts have been identified as commonly featuring appearance focused content [26, 27]. It seems the overwhelming message to adolescent girls is that their value is largely derived from their appearance [28, 29]. Girls can now easily and frequently compare themselves to those they follow on Instagram, whether they are peers or celebrities. The role of social media on body image is also an important issue for consideration among adolescent boys; however, existing research suggests girls are more likely to report negative body image [29, 30].

An increased level of female sexual objectification has been identified through images on social media, where gender inequality is reinforced through the depiction of girls and women as sexually available and objectified [31–34]. Sexual objectification through social media may then lead to adolescent girls’ internalization of conventional ideas of femininity, with subsequent effects on their mental health and wellbeing [34–38]. For body image development, sexually objectified images on social media provide ample opportunity for girls to evaluate themselves against such images which emphasize appearing ‘sexy’ as critical to identity and that their worth is based on constant observation and evaluation of their appearance [36, 39]. Additionally, while masculinity ideals are featuring more frequently in the media, including social media, the sexualization of females remains pervasive compared to males [40].

Previous research conducted on the influence of sexualized media on females’ body image as an indicator of mental health has largely focused on the impact of conventional mass media [41, 42], employed quantitative research methods [21, 34, 43–45], analyzed sexualized content in various forms of media [46], focused on pre/early adolescent girls [29, 47, 48] or young women [32, 49–52]. There are few qualitative studies exploring the influence of sexualized images on social media or the role of social media use in body image development from the perspective of adolescent girls themselves. Of these, the focus has either been on sexualized content only [53, 54], image-sharing practices on social media [31, 33, 55], or the influence of social media use broadly (without a focus on sexualized images) on body image [11, 56, 57]. To the best of the authors’ knowledge, there are no qualitative studies exploring adolescent girls’ perceptions of the influence of sexualized images on social media on their mental health, or body image, as referred to in the present study. Nonetheless, these studies illuminate the ubiquity of appearance-focused and objectified images girls encounter when using social media and the challenges they experience navigating sexualized ideals of femininity [31, 53, 54]. Focus groups with girls and boys found the importance of appearing attractive on social media [33, 55, 56] and the perception that social media negatively impacted one’s body image [57] were more prominent for girls. Additionally, focus groups with only girls reported they frequently use social media to engage in appearance-focused social comparisons and some girls in the study indicated they were dissatisfied with their appearance [11]. These findings, in combination with their limitations related to focus group methodology whereby participants may have provided socially desirable responses, warrant further in-depth exploration with adolescent girls. Therefore, the aim of this study was to explore how sexualized images of females’ bodies typically found on social media might influence adolescent girls’ mental health, in positive and/or negative ways. A generic qualitative approach [58] utilizing in-depth interviews with adolescent girls was used for this study. The findings reported here are part of a broader study that included interviews with parents of adolescent girls, secondary school staff in a support service role such as school psychologists and those on pastoral care teams, and youth mental health service providers. Only the findings from girls are reported in this paper.

## Methods

A generic qualitative research design was used for this study, an approach which is not informed by any one known qualitative methodology and its explicit or established set of philosophical assumptions [58]. A constructivist epistemology [59, 60] guided the study to explore the unique perspectives of adolescent girls using one-on-one in-depth interviews to elicit their thoughts, knowledge and experiences [61, 62].

### Theoretical framework

Objectification Theory has been used to better understand the impacts of being female in a culture that sexually objectifies the female body [34] and suggests this leads to self-objectification whereby females internalize an observer’s perspective as a primary view of themselves and their bodies [63]. Adolescent girls may be particularly susceptible to self-objectification as adolescence is a developmental period of increased self-awareness, self-consciousness, and preoccupation with image and a time when identity is established [64]. When girls encounter sexualized images while using social media, they may self-objectify as they observe and view such content [65]. Additionally, the dual pathway model [66, 67] provides a framework for understanding the mechanisms in which adolescent girls’ social media use can influence their body image. The dual pathway model suggests sociocultural appearance pressures and the internalization of appearance ideals lead to body dissatisfaction and subsequent risk factors for eating disorder development such as disordered eating behaviors [68]. Pressure to conform to appearance ideals through adolescent girls’ social media use and the extent to which they internalize these ideals may contribute to body dissatisfaction [9] and consequently, their likelihood of engaging in disordered eating behaviors with impacts on their mental health [68].

### Participants

A purposive sample of twenty-four adolescent girls aged 14–17 years (Grades 9–11) was recruited for the study from the Student Edge (an Australian student membership organization) youth research panel (n = 13, 54.17%), non-government schools (n = 6, 25%) and through snowball sampling techniques (n = 5, 20.83%) in Perth, Western Australia between 2016 and 2018. Inclusion criteria to participate in the study included active use (i.e., one hour or more per day) of at least one social media platform (i.e., Instagram, Snapchat, Facebook). Most participants were 16–17 years of age (n = 14), spoke English as their first language, and attended a non-government school. One of the participants spoke English as a second language and seven of the participants attended two different all girls’ schools.

### Procedure

Full ethical approval to conduct this research was obtained from the University of Western Australia Human Research Ethics Committee. Student Edge emailed the relevant target audience from their membership base (girls aged 14–17 years in the Perth metropolitan area) and provided a link on their website to a screening survey. The screening survey explained the research project and what participation involved, asked students their age and gender, and if they would like to participate. Those who responded ‘yes’ and met the inclusion criteria for participation (n = 45) had their name, phone number and email address captured based on their Student Edge membership details. These details were then sent to the first author who made contact via phone and/or email to arrange an interview.

To recruit students from non-government schools, approval was first sought from the Catholic Education Office of Western Australia and the Association of Independent Schools of Western Australia then school principals, who were contacted by phone and email seeking their approval for project information to be distributed within their schools via email, newsletter items and flyers. Parents and students were provided with an information sheet describing the research and asked to contact the research team via phone or email if they were interested in participating. School principals were asked to nominate a school-coordinator to assist in arranging student interviews. Additionally, girls were recruited through snowball sampling methods, with those who participated in the study asked to distribute project information to other girls aged between 14 and 17 years.

Prior to participation in the study, written informed consent was obtained from both parents or guardians and adolescent girls. For those recruited from the Student Edge youth research panel, parent or guardian consent was required for students under 15 years to be eligible to become a member.

Adolescent girls participated in one-on-one semi-structured interviews with open-ended questioning undertaken by the first author between October 2016 and February 2018. During the interviews, girls were asked questions in relation to publicly available images of celebrities from Instagram using third person disclosures. These methods were used to enable discussion without participants having to reveal personal experiences which may have caused discomfort, and as a requirement of the study’s ethical approvals. The images were selected from celebrities with some of the largest numbers of female followers on Instagram at the time of data collection (Selena Gomez, Gigi Hadid, Kylie Jenner and Kendall Jenner), and for variation in parts of the body that were emphasized, and the presence of a sexually suggestive pose as used in previous studies [24, 46]. Participants were shown each image and asked what girls their age looking at Instagram might think about the images and why, how the images might make them feel about themselves and why, and how the images might influence mental health (in both positive and negative ways). Participants were also asked for their opinions about the sexualization of girls through images on social media, and in what ways this could be positive or negative. Interviews concluded with asking girls what they thought might help or prevent any of the negative influences on body image they identified. Participants were also asked demographic questions, how often they used social media, and what types of social media they used.

Prior to data collection, the interview protocol was pilot tested with a convenience sample of two adolescent girls aged between 16 and 17 years to provide feedback on question development and types of responses received, as well as the skills of the interviewer. No changes were made to the protocol as a result of the pilot testing.

Ten of the interviews were conducted in person (at their school or a public location) and the remaining fourteen interviews via phone (by participant request). Interviews lasted between thirty minutes and one hour.

### Data analysis

All interviews were audio recorded and professionally transcribed verbatim and imported into qualitative data management software NVivo 11 (QSR International Pty Ltd, 2018) for management, retrieval, and interrogation. Data were analyzed by the first author using thematic analysis as described by Braun and Clarke [69]. This involved immersion in the data through reading and re-reading interview transcripts, followed by the generation of initial codes from features of the data, with some of these forming repeated patterns across the data set. During the initial coding phase, full and equal attention was given to each data item. These codes were then collated into potential themes. Themes were reviewed at the level of the coded extracts to ensure they were coherent, with a candidate thematic ‘map’ created. These themes were then refined to ensure they accurately reflected the data set as a whole, with recoding occurring as required. The thematic ‘map’ of the analysis was then further refined to formulate clear definitions and names for each theme. Throughout analysis the first author discussed the generated codes and themes with the co-authors to ensure accuracy of meaning and interpretation.

The coding frame for thematic analysis included both inductive codes generated from the data itself and deductive codes present in the existing research literature [59]. Codes that did not reflect the data were amended to fit the data. Data were not molded to fit predetermined codes or discarded. To maintain confidentiality, each participant and other entity or institution was allocated a pseudonym during data analysis.

Data collection and analysis procedures were recorded in an audit trail by the first author to document comments, decisions and observations, and to demonstrate and clarify decision-making to ensure any interpretations made accurately reflected the data. This documentation maintains rigor in qualitative research by strengthening the dependability and confirmability of the study [62, 70]. To increase credibility of the research, responses were checked during and on completion of interviews to ensure the representations of participants’ viewpoints were accurate [62].

## Results

As an introduction to participant interviews, adolescent girls were asked about their social media use. These questions related to the different types of social media they used most often, how many hours a day they spent using these (on both a weekday and weekend day), and the device used to access social media.

The most used social media among participants were Instagram, Facebook and Snapchat. An equal number of participants reported they either spent less than two hours, or more than three hours, using social media on a weekday. On a weekend day, most participants spent more than four hours using social media. Delineation between passive use such as scrolling social media app feeds or viewing stories, and active use involving liking, commenting, and sharing posts was not collected as part of this study. Mobile phones were the most commonly used device to access social media. Daily use of social media reported by participants in this study was greater than has been previously reported among Australian females aged between 14 and 24 years, who on average in 2018 spent close to fourteen hours each week, or about two hours per day, on social media [71]. Additionally, time spent on social media by girls in this study is outside of the Australian 24-h movement guidelines for children and young people aged 5–17 years which recommend limiting sedentary recreational screen time to no more than two hours per day [72].

Participants identified body image as a major concern in relation to adolescent girls’ social media use and its influence on mental health, reporting girls felt insecure and self-conscious about their appearance when using Instagram specifically. This was not necessarily related to content participants considered as sexualized. Images were identified as sexualized depending on the amount of skin exposed rather than a females’ pose in an image. Four overarching themes emerged from the data and provided an in-depth understanding of the ways in which the girls in the study described how social media use influences body image: ‘expectation’, ‘comparison’, ‘striving’, and ‘validation’. Participants also referred to ‘counteracting negative body image and influence of social media’. Additional quotes to support each theme described below are included as a supplementary file (see Additional file 1).

### Expectation

Images of other females were perceived to add an expectation for adolescent girls to look a certain way in their own social media posts to obtain what they deem an acceptable number of ‘likes’ and positive commentary. Although this is often influenced by images of celebrities, girls interpreted these as less realistic and attainable, with sexualized images posted by peers and other girls their age having a greater influence on their likelihood to make negative appearance comparisons;

I guess, you know they’re celebrities, so something must have gone into it [a photo]. It’s not just a photo, but I think if it’s someone you know or someone your age, it’s like, “Wow, that really could be me,” or “People my age are looking like this or doing this kind of stuff.” So, I think it would have a worse effect. (Sana, 17 years)

This expectation was perceived to make girls feel pressured to look attractive in their social media posts, even if it meant not being themselves as described in the participant quote below;

Some girls try to look like that [the images shown] and then they’re probably not being themselves, but they’re being what they think they’re expected to be kind of, which is not very good. (Candice, 15 years)

Girls also talked about how expectations experienced from viewing sexualized images on social media would vary between girls, depending on how they already felt about their appearance;

I guess it depends on how the girls feel about themselves first because depending on how they feel about themselves will depend on how they view the photo. (Daisy, 16 years)

I think in general it depends on the mood that you're in when you open your phone. If you're already in a vulnerable mindset or if you've been out all day at the beach or something and you'd come home, you'd probably take more notice of that and be like, "Oh, I wish I looked like that." (Charlotte, 17 years)

While asked about both potential positive and negative influences of sexualized images of females featured within social media, girls could not identify any positives and continually spoke of the negative influences;

I think it would definitely have a negative impact on their mental health because they’d probably really be upset if they can’t achieve those unrealistic body expectations. (Sophie, 17 years)

Expectations related to social media use and body image were also discussed in relation to the normalization of following certain types of Instagram accounts, such as those that are appearance-focused and of attractive females with many followers, and how this could then lead to appearance-based expectations;

I think it [following appearance-focused and popular attractive female Instagram accounts] becomes more accepted and it becomes okay. It’s almost like a visual effect I guess if one particular group of teenage girls follow celebrities or whatever, begin to follow those sort of things [appearance-focused and popular attractive female Instagram accounts] and all people follow them, their friends, it [trying to look like the females in those accounts] becomes more of an expectation. (Brooke, 16 years)

### Comparison

While encouraged to use third person disclosures during interviews, participants reported they made negative appearance comparisons when viewing images on social media. Negative appearance comparisons were made irrespective of whether images were considered sexualized. As in the discussions among girls related to expectation, both images of celebrities and peers influenced comparisons, however, the influence of peers was considered more prolific;

When I see girls my age [on Instagram], I just compare myself to them ‘cause I know it’s kind of reality, if that makes sense, to know that someone my age can look like that and then why don’t I look like that? I think that’s what a lot of girls would see. (Olivia, 16 years)

While images of peers were considered to have a greater influence on negative appearance comparisons among the majority of girls, not all shared this viewpoint;

I think they [girls] would still to a certain extent be like, “Oh, I still want to be them,” but I feel it would be less, because if they see, “Oh, they're just like a regular person, they’re not a celebrity,” then they’re not really worth looking up to. But some people might say, “Oh, I want that kind of life,” for a regular person, like, “Why can she have just such a great life but I don’t?” (Amelia, 16 years)

All four images shown in interviews were perceived by participants to influence girls their age in making negative appearance-based comparisons. Reasons included the celebrities’ current popularity among their age group and the perception that all were attractive. For some participants, the number of likes was considered to play a role in comparisons, with a higher number equating to level of attractiveness. For others, the negative comparison was considered irrespective of the number of ‘likes’. All but one of the images was considered sexualized (where the least amount of skin was exposed), but it was noted that when using Instagram, girls would be unlikely to pause and make this distinction while scrolling through images.

All participants acknowledged the editing behind photos on social media but this did not counteract them making negative appearance comparisons;

A lot of them [photos] are edited and things like that but you don't really think about that when you look at someone's profile, you just compare that to yourself and then, that just makes you feel really bad about yourself. (Emma, 17 years)

Similarly, an awareness of images on social media usually featuring someone at their best did not ameliorate negative comparisons;

‘Cause if they constantly see it – and especially if you’re scrolling, some people might be in bed or on the couch, kind of not looking their best, they compared themselves at maybe their worst, compared to them at their very best and immediately, they go, “Oh, wow, okay.” And they see themselves as so much lower because of the comparison. (Candice, 15 years)

Even when prompted, girls struggled to identify any potential positive comparisons with the images to which they are exposed on social media. Females on social media who post photos of themselves were considered confident and empowered by their appearance, but girls did not agree on whether this would make girls their age feel good about their own appearance.

### Striving

The expectation perceived by participants and the comparisons made from viewing images on social media was seen to influence girls’ striving to look a certain way, portray an enviable lifestyle and obtain many followers, ‘likes’ and comments;

You’re constantly thinking about aspiring to be something that I know 90% of girls aren’t going to be that way. It’s not possible and people need to realize that you’ve got to be happy with who you are and that you're beautiful in your own way. (Matilda, 16 years)

Participants particularly spoke about the influence of images on girls wanting to change their bodies;

You just think, “Oh, that's possible” and then you try and shape your body to be like that, so you eat less and eating disorders occur. (Zoe, 16 years)

Just seeing [images on social media] all the time and it can get you down and girls could think, “Oh, I need to have my body like that.” People are always saying, “Oh, I want to get a summer body,” all the time. (Madeleine, 14 years)

For some girls, fitness accounts on Instagram, in addition to celebrities and peers, were also perceived as influential in girls’ striving to change their bodies;

I think it [images of females on fitness accounts] just puts this really unrealistic vision of what you should look like, and what you should do with your body to girls my age. (Abbey, 17 years)

When discussing the images shown of two popular and attractive models, it was well known among girls that both had been, and were currently, Victoria’s Secret models. This led to considering the type of influence such images have on adolescent girls’ body image;

I do know that a lot of my friends follow [on Instagram] a lot of models and celebrities, especially like Victoria Secret models for instance. I mean I’ve never been into that and that’s just never been my thing but I think that a lot of girls my age are following models. I guess it [is] sort of a way for them to almost, like to see what they aspire to be, which is really sad. (Matilda, 16 years)

### Validation

Intersecting with the themes of expectation, comparison and striving, participants frequently spoke about validation when discussing the influence of social media on body image. A currency of ‘likes’, comments and followers where girls are validated on their Instagram posts and accounts was evident throughout discussions with participants;

I feel that when people post photos of them in their bikini, they want that positive feedback and say, “Oh, you look so amazing.” And that's why they do it because they want the compliments. It's kind of a false representation of themselves because they're just doing it for the likes and the compliments. (Tahlia, 16 years)

This validation was perceived to reinforce to girls that their value is largely placed on their appearance and influenced the types of images they would consider posting of themselves.

Although not frequently identified by participants, some discussed behaviors of possible concern among girls regarding the influence of social media likes, as described by Charlotte (17 years):

If you went to a birthday or something and everyone is eating cake and heaps of food, I think you probably would restrict yourself a little bit more than you would have otherwise. And think, "Oh, they got these many likes and this, maybe I should stop eating a little bit.”

### Counteracting negative body image and influence of social media

Participants discussed the importance of awareness and education from a younger age among girls about social media and its influence on body image. Year six (11–12 years of age) was identified by the girls as an optimal range for this to occur, when girls are starting to use social media and many are experiencing pubertal changes and becoming more aware of their bodies and appearance. Schools, parents, peers and online sources including apps were all perceived by girls as having the potential to play a helpful role in counteracting negative body image messages, particularly when awareness and education can be delivered by all of these sources.

A form of awareness and education commonly identified by girls included critiquing images on social media within the school curriculum, to improve ‘social media literacy’;

Just to be reminded that these things aren’t what they look like. Maybe videos or something that show how edited these photos get. Like, I've seen one and it was about magazine covers, and it was just the beginning of a woman, and then two hours of makeup and things like that later, the end of her. And then she got put on the magazine cover. So, maybe similar things for social media. (Sana, 17 years)

Although it was apparent throughout the interviews with the girls that they were already aware of the editing and enhancement of images on social media, as well as the tendency for images to portray females at their best, they struggled to apply this knowledge. This was especially the case when viewing images of their peers.

It was highlighted that messages to counteract negative body image were needed, including focusing on girls’ strengths rather than their appearance, diversity of physical appearance and that idolized physiques, such as those of celebrities are not the norm;

I think for me the thing that I would like to see is saying yes, this person might be really pretty and this person might not be, but that intelligence and sort of physical [ability] is just as important. I mean, trying to say, “Oh, don’t worry [not] everyone looks good all [the] time.” That’s not helpful ‘cause nobody really believes it. (Brooke, 16 years)

Participants discussed the use of social media to counteract negative body image and promote positive body image, with body positive and acceptance messages including imagery and quotes considered helpful;

There’re a lot of body positive pages, so they post photos of normal people, not like Kendall [Jenner] but people with stretch marks and not like that at all. And then you get quotes and all these amazing things, like people's stories. So you just have to balance it out, I think, which took me awhile to do because, at first, I was just following people like her [Kendall Jenner], which didn't make me feel too good, and then now, I just go half and half. (Ava, 14 years)

While identified by the majority of girls as helpful to counteract negative body image, only a few said they followed such profiles or accounts and some were not aware of any these.

Girl-focused support and programs were discussed as needed to help girls counteract negative body image and the influence of social media, as exemplified by Grace (15 years):

I would just say there needs to be more support directly aimed at girls. I mean just bringing awareness to the fact that social media isn’t the point of your value and your worth, and that people might think that’s stupid but it is really such a big thing and I noticed it with so many people. It’s not the epitome of who you are. There’s way more substance to your person than how many followers you have and just raising awareness and bringing a lot of support and teaching girls self-love and self-worth is important so that you don’t have to have a boy validate that or you don’t have to have ‘likes’ to validate that.

The role of apps in providing girl-focused support was also discussed by participants, although some expressed concern that girls may not seek out such an app;

If it was just like [a] ‘girls only’ app. Like little ways to de-stress. Where you like breathe and stuff like that, I think that it needs to be something like that, but the thing is I don’t know if many girls would use it, I guess. They’d be like, “Why do I need this? This isn’t a necessity for me.” I don’t think many girls know that it’s harmful for them to be comparing themselves to these girls. (Amelia, 16 years)

Both school and other sources such as online environments were identified as settings where such support could be provided. However, girls also stressed the importance of schools not just providing talks about body image or advising them to simply stop engaging with social media that is influencing them negatively, as described in detail by Rachel (17 years):

We have heaps of body image talks, but it’s like, okay, they’re good for the first one, and then they’re sort of repeating themselves and it’s not going in anymore. It’s just your natural instinct to look at someone [and compare yourself]. They’ve told us to go unfollow anyone on Instagram who’s making you upset or whatever. [Its] a lot easier said than done. ‘Cause you don’t really know what’s making you upset. You can be following lots of supermodels and them as a collective are making you upset, but you’re so intrigued on where they’ve got to in their life that you don’t wanna unfollow them.

## Discussion

The current study utilized in-depth interviews to better understand how sexualized images typically found on social media might influence adolescent girls’ mental health, in positive and/or negative ways. Body image was the only aspect of mental health highlighted by participants in this study, attesting to its importance in the minds of participants.

While studies have found sexualized images to influence body image among females [34, 43, 49], participants in this study did not highlight sexualization as a specific concern in relation to body image. The pervasiveness and normalization of sexualized images within social media may help explain why girls participating in this study did not consider such images as distinct from others [34, 52]. However, the four overarching themes of expectation, comparison, striving and validation reported in this study highlighted that adolescent girls largely view their body in relation to their appearance, and suggests self-objectification is a prominent issue when exploring the relationship between social media use and body image. Previous studies have also found a connection between self-objectification on girls’ appearance concerns [40, 51, 52]. Consequently, preventing appearance concerns and negative body image among girls may be facilitated by the development of strategies from a young age to counteract self-objectification, appearance concerns and comparisons in relation to social media use [11, 21, 73].

Consistent with previous research, the influence of social media on adolescent girls’ body image was perceived as negative by the participants in this study [12, 24, 29, 57, 74]. Girls found it difficult to identify positive influences of social media on body image, with little to no discussion among participants, even when prompted during interviews. Participants perceived girls who posted photos of themselves on social media as confident and empowered by their appearance and were unsure whether this would have a positive influence on the body image of other girls their age or those who posted the images. While some existing literature suggests adolescents are unaware and naïve to negative influences associated with social media [2, 23, 75], this study found girls were well aware of how the experiences of expectation, comparison, striving and validation led to negative thoughts and feelings related to their body image. Girls were also able to suggest strategies to counteract negative body image and were able to apply critical thinking when viewing images of celebrities. These findings align with previous research that found adolescents to be critical users and generators of social media, with high media literacy and the ability to identify strategies that may help mitigate social media’s negative effects on body image [11, 76, 77].

Adolescent girls in this study identified the importance of peers in relation to making appearance-based comparisons, with differences in the comparisons made to peers or celebrities, suggesting body image may be more negatively influenced by viewing images of peers on social media. This finding aligns with previous studies identifying peers as having a significant influence on body image concerns among girls [11, 24, 73]. Participants perceived peers as more relatable than celebrities, who they considered as less realistic and attainable. With images on social media more frequently featuring girls’ peers (although images of celebrities are also prominent), this finding adds to existing research highlighting peer appearance comparisons as an important component to address when developing programs aimed at the prevention and early intervention of body dissatisfaction and appearance-based concerns among girls [21, 73, 78]. Additionally, this study found girls were not able to apply critical thinking skills when viewing images of peers, suggesting girls need support to apply these cognitive skills to prevent or minimize peer appearance-related comparisons.

Participants also suggested that some adolescent girls may be more at risk than others of making negative appearance comparisons. This was discussed in relation to how girls already felt about their own appearance and their mood when using social media and viewing images. In relation to how girls already feel about their own appearance, positive body image could play a protective role in influencing the likelihood of making negative appearance comparisons while using social media. Positive body image refers to love and respect of one’s body and emphasizes acceptance and appreciation of its functions irrespective of whether it meets dominant societal appearance ideals [79]. An important characteristic of positive body image pertinent to the influence of girls’ social media use on their body image is protective filtering, whereby positive-body related information is accepted while negative information is rejected, maintaining positive body image [79, 80]. Among a sample of adolescents with positive body image, expressing strong criticism against appearance ideals was found to foster protective filtering and thus helped to uphold positive body image [81], whilst in another study of adolescent girls, protective filtering also suggested benefits to body image [11]. Conversely, a recent qualitative study exploring adolescents’ processing and protective filtering of social media content and perceived protective benefits of these strategies for body image found that although girls in the study displayed aspects of engaging in protective filtering, this did not necessarily translate to protective effects to their body image and they experienced difficulty internalizing positive body-related messages and accepting and appreciating their own bodies [57]. While the present study did not collect data about participants’ own body image, findings support the importance of girls’ varying levels of body image when developing interventions aimed at reducing negative appearance comparisons when using social media.

Participants in this study also considered that a girls’ mood when using social media and viewing images may place some girls at greater risk of making negative appearance comparisons. This finding suggests that body dissatisfaction could be state-based and mediate the influence of viewing images on social media and body image, with the immediate impact of exposure to such images influencing body dissatisfaction. Research conducted with women who had trait-level appearance ideal internalization and body dissatisfaction found appearance comparisons, and in particular upward comparisons (to those deemed more attractive) predicted increased state body dissatisfaction [82]. Adolescent girls who internalize appearance ideals and those with elevated trait body dissatisfaction may be at greater risk of making negative appearance comparisons when using social media and thus may be an important sub-group to consider for intervention. Previous research has also found that girls with higher social comparison tendencies [24] and those focused on gaining approval from others about their appearance, experience more negative effects on their body image as a result of using social media [29]. Gaining approval from others when using social media through ‘likes’ and comments was mentioned frequently among girls in this study and was perceived to provide validation of one’s appearance and thus, reinforcing a focus on appearance. At the time of this study, Instagram had not yet begun its trial of no longer displaying the amount of ‘likes’ on posts. Further research with adolescent girls could explore their views on this change and its influence on appearance-based comparisons and social media activity among this group.

The role of schools, parents, peers and online sources in counteracting negative body image was highlighted by participants in this study, with emphasis placed on body image awareness, education and support being delivered by each of these sources. This finding supports existing research recommending an ecological approach to adolescent body image development, where all interactions in a girls’ environment can be influenced to prevent body dissatisfaction related to social media use [83]. Parents are a key influence on girls’ body image [84, 85], and research has found they can play a protective role in preadolescent and adolescent social media appearance comparisons and body dissatisfaction [86, 87]. Schools provide a setting in which content can be delivered in the classroom and whereby families, peers, teachers and other school staff can be engaged and involved in the implementation of health promotion interventions with a focus on body image [88]. When planning such interventions, it is important to consider girls’ age and developmental stage, as well as the influence and interaction of individual, family, peer, online, community, and school environments on their body image to counteract negative body image.

Congruent with research investigating social media literacy interventions as an emerging approach to address specific challenges to body image posed by social media [89], participants in this study perceived improved social media literacy among adolescent girls from a younger age, taught within the school curriculum, as important to counteracting negative body image. Social media literacy focuses on the interactions among users of social media, whether friends, other peers or celebrities, as well as developing the skills to examine the messages underlying commercial media advertising, including health and fitness, seen on social media [78]. This finding aligns with previous research which has observed favorable effects on body dissatisfaction, internalization of the thin ideal, appearance comparison, and self-esteem among girls following a pilot social media literacy intervention adapted from the ‘Happy Being Me’ program [90]. However, a recent randomized controlled trial found less effectiveness as a stand-alone intervention, with the appearance-comparison component found to be more effective [78]. Participants in the present study also identified appearance-based comparisons as a topic of concern to them, suggesting the need to include both social media literacy and appearance-comparison content in body dissatisfaction prevention interventions.

When discussing strategies for counteracting negative body image and the influence of social media, participants also referred to the importance of promoting positive body image through messaging focused on girls’ strengths rather than their appearance, body acceptance and ways to challenge unrealistic societal appearance ideals. This finding aligns with sociocultural theories such as the dual pathway model [68] suggesting the pressure among girls to conform to appearance ideals and the extent to which they internalize such ideals are important factors to target in interventions aimed at this group. To this effect, cognitive dissonance intervention the Body Project has a strong body of evidence supporting its effectiveness in increasing body appreciation and reducing thin-ideal internalization and body dissatisfaction among adolescent girls when implemented in schools [91–95]. The theoretical premise of the Body Project is that when there is a discrepancy between an individual’s beliefs and actions, they experience discomfort i.e. cognitive dissonance, which they then try to avoid, becoming motivated to re-assess their beliefs to align with their actions [96]. In the intervention, this is facilitated by group discussions and activities with adolescent girls where girls actively challenge appearance ideals with subsequent decreases in thin-ideal internalization and body dissatisfaction [91]. Additionally, research indicates acceptability of the intervention among adolescent girls, with the group setting contributing to their sense of belonging, particularly when facilitators are considered relatable, such as undergraduate female university students [95, 97].

The finding that any negative influence of social media on body image was not necessarily in relation to sexualized content highlights the importance of undertaking research with girls to better understand the mechanisms of social media’s influence on their body image. In this study, participants made negative comparisons with images of females on social media regardless of whether they were considered sexualized, with the influence of peer appearance comparisons more prominent. Research with adolescent girls will also enable them to inform and co-develop interventions to support their body image development and prevent or reduce harms experienced from their social media use in relation to body image, targeted to the needs and interests of their age group.

The current study contributes new knowledge from the perspective of adolescent girls to the existing literature on adolescent girls’ social media use and its influence on their body image. The findings of this study suggest that social media can have a negative influence on girls’ body image through negative appearance comparisons when viewing images on social media, exacerbating appearance-based concerns and body dissatisfaction. While negative comparisons were made irrespective of whether images were considered sexualized, findings suggested a level of self-objectification among adolescent girls whereby they viewed themselves in relation to their appearance. The important role of peers in appearance comparisons was also evident in this study. Participants also identified strategies to prevent and counteract negative body image, which have important implications for the development of health promotion programs addressing social media use and body image concerns among adolescent girls for prevention and early intervention that can minimize potential harms. For parents and professionals working with adolescent girls, particularly in the school setting, the findings can be applied in their work by providing education about social media and its influence on body image and strategies to prevent and counteract negative body image to support girls.

### Limitations

This study has several limitations that should be considered when interpreting its findings. This study was exploratory and limited by a small number of self-selected participants (n = 24). Therefore, its findings cannot be used to make assumptions about the population of girls aged between 14 and 17 years in Perth, Western Australia and does not claim to be representative of the broader population of girls. Findings may vary in other areas of Western Australia, Australia and internationally. However, qualitative research often uses smaller samples enabling the collection of in-depth information and providing direction for further research.

Additionally, participants’ own body image concerns/body dissatisfaction were not assessed as part of this study. The participating girls’ feelings about their body image may have influenced their perceptions of how social media influences body image among other girls.

The interpretation of this study’s findings may also be influenced by the characteristics of the participating girls. There were slightly more participants in this study aged between 16–17 years old, and these girls may have been using social media for longer compared to younger participants. Age and more years of experience using social media may have influenced participants’ interest in issues related to social media and thus their interest in participating in the study. In addition, all but one of the girls were from an English-speaking background and findings may differ among girls from culturally and linguistically diverse backgrounds, as they may not feel they meet Western appearance ideals and may also experience different perceived sociocultural appearance-related pressures depending on their cultural background. Another limitation of this study was that most participants attended non-government and co-educational schools. It is possible that findings may be different among samples where girls largely attend government or all girls’ schools. As most participants attended non-government schools and were from higher socioeconomic backgrounds, they may have had increased access to digital technology and therefore use of social media. Additionally, girls from high socioeconomic backgrounds may experience differences in perceived appearance ideals compared to girls from different backgrounds. It would be useful for future research to explore further the perceptions of girls in government schools and all girls’ schools to allow for comparisons, especially in relation to peers and sexualized images with those in non-government and co-educational schools.

## Conclusion

This study provides some insight into the influence of social media on adolescent girls’ body image from the perspective of girls in Perth, Western Australia. Further research should engage with adolescent girls to identify and investigate the impact of strategies to prevent and counteract negative body image related to social media utilizing an ecological approach to encompass all aspects of girls’ lives.

## Supplementary Information

**Additional file 1**. Thematic table illustrating additional quotes from interview findings.

## Data Availability

The datasets generated and/or analyzed during the current study are not publicly available to protect the anonymity and confidentiality of the participants. Requests to obtain datasets can be made to the corresponding author.
