# Supplementary material for: “Why don’t I look like her?” How adolescent girls view social media and its connection to body image
Source: BMC Womens Health. 2022 Jun 27;22:261. doi: 10.1186/s12905-022-01845-4 (PMC9238066; doi:10.1186/s12905-022-01845-4)
Supplement: Supplementary file 1 — Additional file 1. Thematic table illustrating additional quotes from interview findings. [file 12905_2022_1845_MOESM1_ESM.docx]

Additional file 1

Thematic table illustrating additional quotes from interview findings

| Theme | Additional quotes |
| --- | --- |
| Theme 1: Expectation | I just think that girls my age are so caught up on the way that they look, and they have so much pressure on them that I just don’t think is necessary at this age or any age. I don’t think any person of any age [needs] to be pressured into thinking they don’t look good enough. (Matilda, 16 years) |
|  | I mean I think when anyone sees a picture like that [those shown in interview], it sets the bar higher for everyone else because like, “Oh, look at all this work they put into the picture.” Everyone else also has to meet that standard. (Brooke, 16 years) |
|  | It could be something to look at and see as a role model [female in image shown in interview], but it's not a role model. It's an expectation. (Gabrielle, 14 years) |
|  | They [girls] think that they’ve got to look like that [images shown in interview] to feel they’re pretty or something, and that that’s what you have to be. (Jasmine, 14 years) |
| Theme 2: Comparison | I guess you could just see it as an empowering thing that women are feeling comfortable in their bodies, but it just isn’t the first thing that comes up. It’s more, “Why don’t I look like her,” type thing. (Matilda, 16 years) |
|  | They [girls] might go, “Oh, I don’t look like that and I should look like that [when viewing images on Instagram].” Yeah, and think “I’m nothing like that. I’m not pretty and I’m not confident,” Comparing themselves. (Candice, 15 years) |
|  | I know a lot of people do struggle, especially a lot of girls have eating disorders, it’s horrible. I think probably it is influenced because you're looking at people [on Instagram] and you're like, “Oh, why does she eat that much and is so thin? Why do I eat this and I'm not thin?” (Isla, 17 years) |
| Theme 3: Striving | I think they [girls] would just think really badly of themselves [when looking at the images shown in interviews]. I know this from personal experience with one of my friends. She started going on this diet because she wanted to be like these Victoria’s Secret models. She went on a diet and everything, she got super skinny, but she still felt really insecure about her body. (Amelia, 16 years) |
|  | They [girls] compare themselves to those photos [such as those shown in interviews] and wanna look like them and feel like they have to stop eating to be their weight. (Julia, 14 years) |
|  | Other girls would find them [images shown in interviews] pretty and then that would affect them through the way of trying to improve their self, their body, when they see those pictures. (Lucy, 14 years) |
|  | I think that mainly the Kendall Jenner one [of the images shown in interview], I think that they [girls] would just compare themselves, like, they would think “Why don’t I have a body like that?” and that would be their goal. They’d want to look like that and then just start shaming what they look like. (Madeleine, 14 years) |
| Theme 4: Validation | So if it [an image a girl has posted of herself on Instagram] got double as many [likes] as another, I think the one with the most likes, you look at that [and] you'd be like, "Oh maybe I need to try and look like that." (Charlotte, 17 years) |
|  | The likes and comments [on Instagram], I think they [girls] rely on them for self-confidence. (Lucy, 14 years) |
|  | I know sometimes if I'm going out and I do my makeup, I kind of feel the urge to post it on social media [for feedback]. (Abbey, 17 years) |
|  | I guess when people comment [on images posted on Instagram] and they're like, "Oh you look great," I guess that's kind of positive reinforcement. (Charlotte, 17 years) |
| Theme 5: Counteracting negative body image | They [girls] just need to understand that you come in different shapes and sizes. At a young age, you're going through puberty, and you don't understand what's happening to your body. (Tahlia, 16 years) |
|  | Talk to girls [at school] about how you don’t need to look a certain way. Everyone has their own body weight that they’re supposed to have. Everyone has a body type. They shouldn’t have to lose weight. (Julia, 14 years) |
|  | I think they [school] should talk about it [body image and the influence of social media] so much more. We talk about other things that aren’t as important as that. I feel like a lot of people need to learn about how to love yourself and take care of yourself and love your body. (Madeleine, 14 years) |
|  | I definitely think that critiquing social media, analyzing it, and breaking it down like you would a literature book would definitely take a lot of pressure off girls. (Matilda, 16 years) |
